# Case Report: Hepatic Sarcoid-Like Reaction Associated With Checkpoint Inhibition in a NSCLC Patient and a Literature Review

**DOI:** 10.3389/fonc.2022.824308

**Published:** 2022-03-10

**Authors:** Yuxin Lin, Wei Zhu, Bingchen Wu, Huiyin Lan

**Affiliations:** ^1^ Department of Oncology, Hospital of Chinese Medicine of Changxing County, Huzhou, China; ^2^ Department of Thoracic Radiation Oncology, Cancer Hospital of the University of Chinese Academy of Sciences (Zhejiang Cancer Hospital), Hangzhou, China; ^3^ Institute of Cancer and Basic Medicine, Chinese Academy of Sciences, Hangzhou, China

**Keywords:** Sarcoid-like reaction, immune checkpoint inhibitor, Toripalimab, PD-1, case report

## Abstract

Immune checkpoint inhibitor (ICI) treatment has dramatically revolutionized the landscape of therapeutic approaches in multiple cancers, particularly, non-small-cell lung cancer (NSCLC). With the increasing use of programmed death-1 (PD-1) inhibitors in the clinic, the emerging toxicity profile presents a novel learning curve for clinicians. Here we report the first case of an NSCLC patient displaying sarcoid/granulomatous-like reaction (SLR, also known as GLR) in the liver during an anti-PD-1 therapy which showed efficacious response of complete regression. Also, this is the first report describing the SLR induced by toripalimab, a novel PD-1 inhibitor. Given this kind of hepatic findings can be easily mistaken as metastasis, even resulting in premature use of second-line treatments. In particular, we briefly review the clinical features of all those cases reporting sarcoidosis and SLRs manifested on different organs during anti-PD-(L)1 therapy. We anticipate that these clinical cases would help to alert the attention of clinicians that SLRs, as a rare immune-related adverse event (irAE), is manageable and that histopathological analysis is necessary before interpreting it as disease progression.

## Introduction

Lung cancer represents the most commonly diagnosed neoplasm and the leading cause of cancer-related death worldwide, and approximately 85% of the patients are histologically categorized as NSCLC (1). Although the introduction of targeted therapies has largely improved outcomes of NSCLC patients, heterogeneity of responses and resistance to these agents are noted. Given the fact that most of the patients have advanced disease on presentation, the 5-year survival among all NSCLC patients remains less than 18% (2, 3). Recently, ICI treatment has become a groundbreaking change in cancer management, as it may achieve enormous long-term survival benefits in several cancers, particularly, NSCLC. Based on the marvelous results achieved in a series of clinical trials, pembrolizumab (an anti-PD-1 antibody) and atezolizumab (an anti-PD-L1 antibody) have already gained global approval as first-line options for eligible patients with metastatic NSCLC either as monotherapy or combined with chemotherapy (4–9). In fact, more and more novel agents targeting PD-(L)1 are continually under investigation. Toripalimab is such a selective recombinant, humanized immunoglobulin G4 monoclonal antibody against PD-1. After having received its global approval in China for use in the unresectable or metastatic melanoma that has failed previous systemic treatment, several clinical trials of toripalimab in other advanced/metastatic cancers are underway (10). Most recently, a phase I trial reported that toripalimab displayed encouraging antitumor activity and manageable safety profiles in NSCLC patients, which is comparable to other PD-1 antagonists such as nivolumab and pembrolizumab (11).

However, along with the robust antitumor activity displayed, ICI treatment also causes non-specific systemic consequences in the setting of immune activation. Specifically, toxicities manifest as a wide spectrum of immune-related adverse events (irAEs), namely, dermatitis, endocrinopathies, autoimmune colitis, pneumonia, hepatitis, and neuropathies (12). Sarcoidosis or SLR is a granulomatous (like) disease which has been sporadically reported and recognized as a rare, but important, kind of irAE secondary to ICI treatment (13). In this present article, we report the first case of an NSCLC patient who got a CR on toripalimab treatment and displayed the hepatic SLR mimicking disease progression. In addition, we also summarize the recent pieces of literature that describe sarcoidosis and SLR induced by ICI therapy in various cancers.

We present the following case in accordance with the CARE reporting checklist.

## Case Presentation

**Figure 1:**
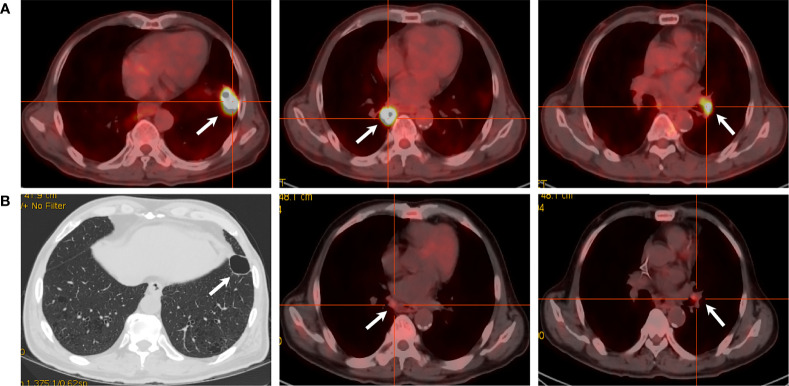
PET/CT scans of the target lesions. **(A)** Before toripalimab and chemotherapy administration in February 2020, PET/CT showed three lesions with intense hypermetabolic activity. **(B)** After 4 cycles of the combination treatment in June 2020, the solid portion of the left lower lobe lung mass disappeared with a cavity left. Also note the dramatic decrease in the size and metabolism of the other two masses.

A 69-year-old Chinese man was diagnosed with squamous cell lung carcinoma in February 2020, complaining of stimulating dry cough for 10 months and tachypnea for 2 weeks. Chest and abdomen contrast enhanced computed tomography (CT) scan showed two masses in the bilateral lower lung lobe (right: 32 mm, left: 31 mm) and the appearance of left hilar lymphadenopathy. A CT-guided percutaneous intrathoracic lung biopsy was conducted to the mass on the left lower lung lobe and a histopathological analysis confirmed it as squamous cell lung carcinoma. In addition, both of the pulmonary masses and left hilar lymphadenopathy were confirmed as hypermetabolic on [^18^F]2-fluoro-2-deoxy-D-glucose–positron emission tomography/CT (FDG–PET/CT) (**Figure 1A**). Brain magnetic resonance imaging (MRI), bone scan, and abdomen B-ultrasound were performed and showed no metastatic findings. Given these clinical contents, he had a final diagnosis of metastatic squamous cell lung carcinoma with stage IVA (cT2N1M1a) which is unresectable. The immunohistochemistry (IHC) result of the biopsy from the tumor tissue showed the positive expression of PD-L1 protein (PD-L1 >1%, TPS, 22C3). After multidiscipline discussion and communication with the patient, he received PC chemotherapy regimens (paclitaxel 300 mg and carboplatin 400 mg), along with toripalimab (240 mg) at day 1 every 3 weeks. The response of the patient was remarkable: a routine CT scan after two cycles of this combination treatment showed about 80% of tumor shrinkage of the known tumoral masses and hilar lymphadenopathy; a repeated CT scan after 4 cycles showed that all the lesions have almost disappeared and the amount of FDG uptake were lost from the PET/CT scan (**Figure 1B**), with an evaluation of clinical CR by the Response Evaluation Criteria in Solid Tumors (RECIST) version 1.1.

**Figure 2:**
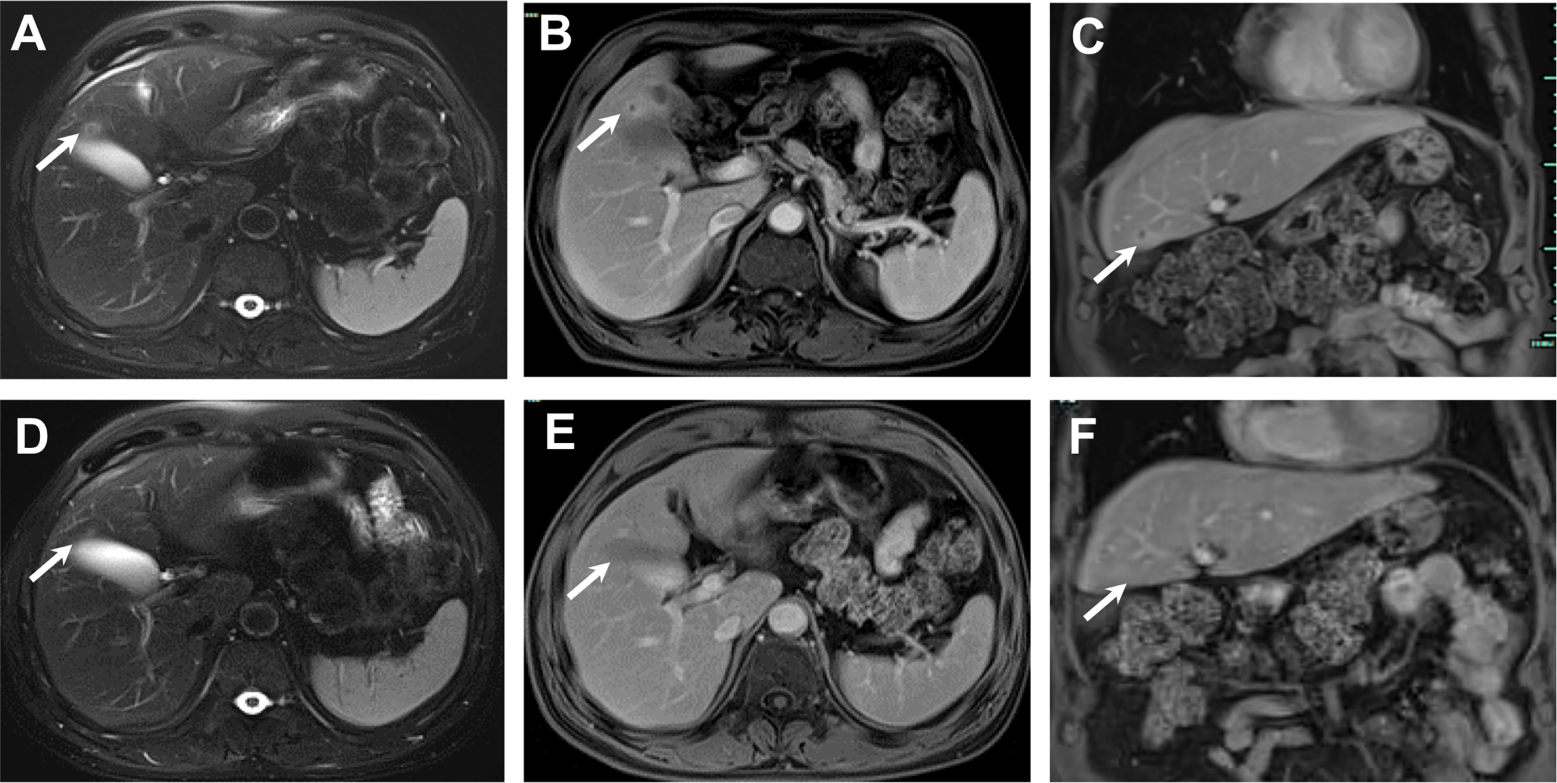
The MRI course of the SLR in liver. November 2020, 13 mm sized nodule in liver quadrate lobe showed circular hyperintensity on axial unenhanced T2-weighted image **(A)** and circular hyperenhancement in portal venous phase with axial image **(B)** and coronal image **(C)**. January 2021, post 2 more doses of toripalimab, the nodule in liver quadrate lobe shrunk to 6 mm in diameter, axial unenhanced T2-weighted image **(D)** and axial enhanced T1-weighted image in portal venous phase with axial image **(E)** and coronal image **(F)**.

**Figure 3:**
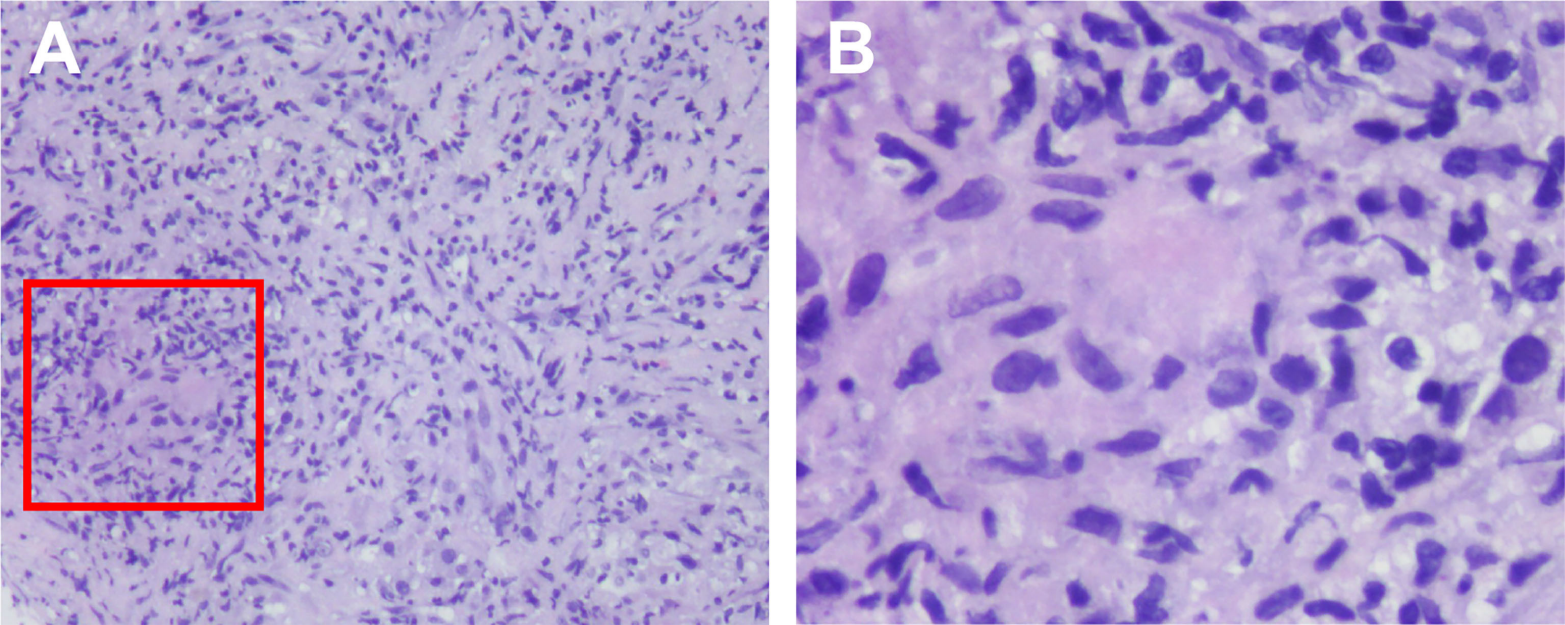
Histological findings of the SLR in liver. Histologic examination of the biopsy tissue showing noncaseating epithelioid granulomatous reaction (within the red box) by hematoxylin–eosin stain, with a mixed cellular infiltrate of lymphocytes, macrophages and fibrocytes. The magnification were ×10 **(A)** and ×40 **(B)**.

Given the favorable tumor regression, chemotherapy of PC regimens were discontinued after 4 cycles and toripalimab as a single drug treatment was continued. Overall, toripalimab was well tolerated except that of a cutaneous diffuse maculopapular rash with mild itching which was improved dramatically and kept on a good control with the treatment of oral antihistamine agents plus topical compound flumetasone ointment. However, a CT scan indicated a 10.0 × 6.3 mm hypodense nodule in the liver quadrate lobe after 6 cycles, which was considered as disease progression with live metastasis in support of the radiology examination, namely, abdominal contrast enhanced CT, hepatic ultrasound, and hepatic contrast enhanced MRI (**Figures 2A–C**). A radiofrequency ablation of liver metastasis and a secondary systemic chemotherapy had even been placed on the agenda. The clinical presentation of the patient, however, seemed to be in healthy status without any symptoms or liver enzyme elevation. Specifically, acid-fast and periodic acid-Schiff-diastase staining was negative for atypical fungal and mycobacterial infection. The patient denied any preexisting granulomatous disease and a thorough evaluation of virus, mycobacterium tuberculosis, autoantibodies were all negative. Laboratory tests of aspartate aminotransferase (AST), alanine transaminase (ALT), γ-glutamyl transpeptidase (GGT), alkaline phosphatase (AKP), bilirubin, and serum calcium were within normal range. Considering the paradox that remote metastasis occurs while primary tumors are still kept in good control, and the discrepancy between the radiology findings and clinical presentation, an ultrasound-guided biopsy of the liver nodule was conducted to ensure a correct diagnosis. Histopathological analysis revealed a noncaseating granulomatous pattern with a mixed cellular infiltrate of lymphocytes, macrophages, and fibrocytes, but no malignant cells (**Figures 3A, B**). Taken together, the patient was referred as a granulomatous disease induced by immunotherapy, and hepatic metastasis was ruled out. Given no other clinical features of multi-organ involvement, such as mediastinal lymph node enlargement, were observed in this case, a final diagnosis of SLR was made for this patient. As the patient was systemically in excellent health, he was continued with the immunotherapy and no immunosuppressive treatment towards the hepatic SLR was administered. After 8 cycles of toripalimab treatment, radiographic evaluations showed that the hepatic nodule had almost disappeared spontaneously (**Figures 2D–F**) and the primary malignancies were still in CR. Up until October 25, 2021, this patient had received 23 cycles of toripalimab treatment (4 cycles of PC chemotherapy along with toripalimab and 19 cycles of toripalimab alone maintenance immunotherapy), and he is well-tolerated in good health with sustained CR of the primary intrathoracic lesions.

## Overview of the Clinical Features of Sarcoidosis and SLRs Associated to ICIs Treatment

SLRs refer to the atypical response patterns of localized non-caseating granulomatous inflammation without fulfilling the systemic sarcoidosis criteria. Unlike the typical sarcoidosis which is obscure in etiology, SLR is majorly described in the setting of active cancer or after the delivery of immunotherapy (14). It was reported that PD-1 or PD-L1 expression was increased both in sarcoidosis and in the peripheral blood upon PD-1 blockade treatment, which may contribute to the pathogenesis of SLR (15–17). Although the exact underlying mechanism remains elusive, a line of cell factors such as interleukin-2, interleukin-12, interferon-γ, and tumor-necrosis factor-α is thought to be involved in the formation of granulomatous inflammation (14, 18, 19). To better understand the clinical presentation of this rare irAE, we briefly summarize all the searchable studies reporting sarcoidosis and SLRs related to ICIs therapy published in the English literature up to February 2021. We identified a total of 53 cases developing sarcoidosis or SLRs, which were summarized in **Table 1**, with the content of demographic features, primary malignancy, ICI treatment, sites, onset and follow-up.

**Table 1:** Published cases of sarcoidosis-like reactions induced by immunotherapy.

Sex/Age	Type of cancer	Type of ICIs	Onset time	Sites of SLR	Treatment of SLR^#^	ICIs withhold	Status	Ref
M/44	Melanoma	Ipili	18 m	Spleen	None	No	PR	(20)
M/38	Melanoma	Ipili	7 m	CNS, LN	Prednisolone	Yes*	SD	(21)
F/46	Melanoma	Ipili	3 m	Skin, lung	Topical clobetasol, oral corticosteroids	Yes	PD	(22)
F/52	Melanoma	Ipili	1 m	Spleen, lung, LN	Methylprednisolone	Yes	SD	(23)
M/55	Melanoma	Ipili	1 m	Skin, lung, LN	Prednisone	Yes*	PD	(24)
M/31	Melanoma	Ipili	43 m	LN	None	Yes*	PD	(25)
M/63	Melanoma	Ipili	5 m	LN	None	Yes*	CR	(26)
F/66	Melanoma	Ipili (NAT)	2 m	Spleen, lung, LN	None	Yes	RF	(27)
F/44	Melanoma	Ipili (NAT)	3 m	LN	None	Yes*	RF	(28)
M/57	Melanoma	Ipili (NAT)	1 m	Skin, LN	Oral prednisone	NR	RF	(29)
M/41	Melanoma	Ipili (NAT)	2 m	Spleen, LN	None	Yes	RF	(29)
F/57	Melanoma	Ipili,pembro	8 m	Skin, lung, LN	Dexamethasone	Yes	PD	(30)
M/50	Melanoma	Ipili,pembro	9 m	LN	NR	NR	GR	(31)
M/69	Melanoma	Pembro	6 m	LN	None	No	CR	(28)
F/43	Melanoma	Pembro	5 m	Skin, LN	None	Yes	NR	(29)
F/50	Melanoma	Pembro	4 m	Spleen, LN, pancreas, bone	None	Yes	GR	(32)
F/69	Melanoma	Pembro	1 m	Skin, lung, LN	NR	NR	NR	(33)
F/58	Melanoma	Pembro	12 m	Bone, skin, LN	Prednisone	Yes	CR	(34)
F/66	Melanoma	Pembro	3 m	Skin, LN	Prednisolone	NR	GR	(31)
M/64	Melanoma	Pembro	12 m	Bone, skin, lung, LN	None	Yes	CR	(35)
M/51	Melanoma	Pembro	9 m	LN	NR	NR	GR	(31)
M/64	Melanoma	Pembro	2 m	Skin	None	No	NR	(36)
F/62	Melanoma	Pembro	2 m	Skin, lung, LN	None	Yes	NR	(37)
F/69	Melanoma	Pembro	1 m	Skin, lung, LN	Prednisolone	Yes	NR	(37)
F/71	Melanoma	Pembro	4 m	Skin	NR	NR	CR	(38)
F/83	Melanoma	Pembro	11 m	Uvea, skin, LN	None	Yes	CR	(39)
F/68	Melanoma	Pembro	6 m	Choroid, LN	None	No	NR	(40)
M/78	Melanoma	Pembro	18 m	Skin, soft tissue, LN	None	Yes	CR	(41)
M/60	Melanoma	Pembro	12 m	Skin, lung	NR	Yes*	CR	(42)
M/81	Melanoma	Pembro	18 m	Skin, LN	Intralesional triamcinolone	Yes	CR	(28)
M/45	Melanoma	Nivo	2 m	Skin, LN	Prednisolone	Yes	NR	(37)
F/56	Melanoma	Nivo	4 m	Skin	NR	NR	NR	(43)
F/69	Melanoma	Nivo	2 m	Skin, LN	Naproxen	No	PD	(44)
M/59	Melanoma	Nivo	10 m	Skin, LN	None	Yes	CR	(45)
M/69	Melanoma	Nivo	3 m	Lung, eye	Oral prednisolone	Yes	PR	(46)
M/66	Melanoma	Nivo (NAT)	4 m	Skin	NR	NR	NR	(47)
M/55	Melanoma	Nivo (NAT)	3 m	Choroid, lung, LN	None	Yes	RF	(48)
M/71	Melanoma	Nivo	5 m	LN	Prednisone	Yes	SD	(25)
M/59	Melanoma	Nivo	0.75 m	Lung	Oral prednisone	No	PD	(49)
M/57	Melanoma	Nivo (NAT)	3 m	Bone, LN	None	No	RF	(50)
F/53	Melanoma	Nivo (NAT)	2 m	Skin, lung, LN	Methylprednisolone, prednisone	Yes	RF	(51)
M/32	Melanoma	Nivo (NAT)	4 m	LN	Prednisone	Yes	RF	(25)
M/57	Melanoma	Nivo/ipili	3.2 m	LN	Oral hydrocortisone	No	RF	(18)
F/64	Melanoma	Nivo/ipili (NAT)	9 m	LN	Prednisone	Yes*	RF	(25)
F/50	Melanoma	Nivo/ipili	3 m	Skin, LN	Oral prednisolone	Yes*	PR	(52)
F/55	Melanoma	Nivo/ipili	1 m	Skin, lung, LN	Prednisone	Yes	PR	(30)
M/68	Melanoma	Nivo/ipili	11 m	CNS	Dexamethasone, infliximab,methotrexate	Yes*	SD	(53)
			5 m	LN	None	Yes*	SD	
M/67	Melanoma	Nivo/ipili	1 m	LN	None	Yes	SD	(54)
			12 m	CNS	Dexamethasone, methylprednisolone, infliximab, methotrexate	Yes*	SD	
M/63	Melanoma	Pembro/ipili	24 m	LN	None	Yes	SD	(25)
M/74	NSCLC	Pembro	4 m	LN	None	NR	PR	(55)
M/55	NSCLC	Pembro	4 m	Lung	NR	NR	NR	(56)
M/69	NSCLC	Nivo	2 m	LN	None	No	PR	(57)
M/76	NSCLC	Nivo	1 m	Lung, LN	None	No	CR	(58)
F/63	NSCLC	Nivo	3 m	Skin	Methylprednisolon,prednisone,Hydroxychloroquine	Yes	SD	(15)
F/56	NSCLC	Nivo	3 m	LN	None	Yes	PR	(17)
M/81	NSCLC	Nivo	3 m	Lung	None	Yes	PR	(59)
F/76	NSCLC	Durva (NAT)	3 m	LN	Oral prednisolone	Yes*	PD	(60)
F/50	BC	Atezo	3 m	Lung, LN	NR	NR	SD	(61)
F/69	BC	Atezo	2 m	LN	None	No	CR	(62)
F/77	Urothelial cancer	Pembro	0.75 m	Skin	NR	NR	NR	(47)
M/52	Urothelial cancer	Nivo/ipili	2 m	Skin, LN	Plaquenil, Methylprednisolone	Yes	SD	(63)
F/64	Renal carcinoma	Nivo	10 m	LN	NR	NR	NR	(64)
F/58	uLMS	Pembro	2 m	Lung, LN	None	Yes	PD	(65)

^#^Treatment of SLR: Prednisolone, prednisone or methylprednisolone without indicated as “oral or topical” was used by intravenous.

*Yes: It refers to those patients with ICIs discontinuation, not because of sarcoidosis or SLRs, but due to events including other treatment schedules, other AEs or disease progression.

Age, age at sarcoidosis diagnosis; Atezo, atezolizumab; BC, breast cancer; CNS, central nervous system; CR, complete remission; GR, good response; F, female; ipili, ipilimumab; LN, lymphadenopathy; M, male; m, months; NAT, neoadjuvant therapy; Nivo, nivolumab; NR, not reported; NSCLC, non-small cell lung cancer; PD, progressive disease; PR, partial remission; Pembro, pembrolizumab; SD, stable disease; uLMS, Uterine leiomyosarcoma.

### Demographic and Epidemiologic Features

Based on these records, the median age at the onset of SLRs was 60 years (range, 31–83 years), with a comparable male to female ratio (34/63, 54%). The most common primary tumor was melanoma (49/63, 78%), followed by NSCLC (7/63, 11%), and others. Most cases were documented with treatment of PD-1 inhibitors nivolumab (18/63, 29%) or pembrolizumab (22/63, 36%), followed by CTLA4 inhibitor ipilimumab (12/63, 19%), the combination of CTLA4 and PD-1 inhibitor therapy (7/63, 11%), and anti-PD-L1 antibodies atezolizumab/durvalumab (2/63, 3%). Toripalimab, however, has not been reported so far. A total of 52 cases (83%) occurred in the setting of palliative therapy for metastatic diseases, and 11 cases (18%) in neoadjuvant therapy. The onset time ranges from less than one month to 43 months after immunotherapy initiation, with a median interval of 3 months.

### Clinical Phenotypes, Symptoms and Diagnosis

In regards with the clinical phenotypes, multi-system involvement occurred more frequently than single organ involvement (58.0% vs 42.0%). The thorax (intrapulmonary, hilar and mediastinal lesions) and skin (subcutaneous and cutaneous lesions) were two most commonly affected sites, with 84.1 and 42.9% respectively. Other organ involvement, such as extra-thoracic lymphadenopathy, spleen, central nervous system, eyes and bone were relatively rare.

As for the accompanied symptoms, subcutaneous and cutaneous reactions usually presented as cutaneous erythema, subcutaneous nodules with itching or pain. A few cases manifested with nonspecific flu-like symptoms such as arthralgia, myalgia, fatigue, fever, sweat, chill, nausea, and vomiting. When central neural system (CNS) was implicated, symptoms of headache, aphasia, visual field deficits, and seizure would develop. These symptoms were always in a mild to moderate extent. But life-threatening ones occasionally occurred with a relative higher incidence among patients with neural and intrapulmonary implications.

The diagnosis of sarcoidosis and SLRs is usually challenging, given a variety of cases were asymptomatic and the nodular lesion was only revealed by radiological evaluations. It is difficult to distinguish between metastatic and granulomatous lesions by both contrast enhanced CT and MRI (27). Neither can FDG-PET/CT, since FDG uptake is associated with enhanced cellular metabolism but irrespective of the nature of the cells. Therefore, it is predisposed to draw a false positive report for a malignancy (66), and differential diagnosis is crucial. Misdiagnosing SLRs as progressive diseases is apt to misdirect the course of cancer management. For example, in the case reported by Swathi B. Reddy, a melanoma patient developed mediastinal/hilar lymphadenopathy and several subcutaneous nodules after 8 cycles of ipilimumab monotherapy. Those newly occurred lesions were firstly referred as disease progression and secondary pembrolizumab was initiated. Her condition worsened dramatically after one dose of pembrolizumab with fever, dyspnea, nausea, vomiting, and transaminitis. A chest CT scan showed emerging bilateral pulmonary consolidations. A biopsy was taken and a histopathology examination revealed non-necrotizing granulomatous inflammation consistent with sarcoidosis. Subsequently, pembrolizumab was withheld, corticosteroid therapy was initiated and this patient generally recovered from this irAE (30). A similar scenario was also seen in other cases (37, 46). In particular, a special ^68^Ga-DOTA-NOC/TATE PET/CT, adopted by Anne-Leen Deleu and her colleagues, was reported to be capable of distinguishing atezolizumab-induced thoracic SLRs from malignancies in a triple-negative breast carcinoma patient (61). However, further exploration for diagnosis is absolutely warranted, and in this case, tissue biopsy is still the gold standard for diagnosis nowadays.

### Treatments

The managements to this adverse side effect were quite personalized. Watchful waiting was chosen in 11 cases without any medical intervention because of limited organ affection and mild symptoms. Corticosteroids were the mainstay of immunosuppressive therapy when necessary. The cessation of immunotherapy and the admission of corticosteroids were adopted in 68% (23/40) and 49% (25/51) of cases, respectively. Appropriate corticosteroid treatments were efficient for symptom relief and may not attenuate anti-tumor efficacy of immunotherapy. Tropical, oral or intravenous corticosteroids were prescribed according to the manifestations, which worked well in most cases (51). In a few refractory cases, infliximab, methotrexate, mycophenolate, and hydroxychloroquine were also considered (15, 53, 54). Overall, ICI-induced sarcoidosis and SLRs were manageable with benign outcomes. Either resolution or improvement was noted in 89% (47/53) of reported cases, and the rest of them are stable. After good control of sarcoidosis and SLRs, the reintroduction of immunotherapy was also considered in some cases. Interestingly, it seems like patients with this kind of documented irAEs preferred better clinical benefits on immunotherapy. Clinical response rate was documented for 54% (25/41) of metastatic patients, followed by stable disease with 22% (9/41), while only 17% (7/41) of patients experienced tumor progression, highlighting the potential role of this reaction as a predictive parameter to ICI therapies (28, 47).

## Discussion and Conclusion

Immunotherapy, especially the checkpoint inhibitor-based immunotherapy, has been a major breakthrough in the field of oncology for providing an efficacious and durable therapeutic option for patients with advanced-stage cancer. PD-1 antagonists selectively block the PD-1 receptor and attenuate its interaction with the ligands, PD-L1, and PD-L2 (67). Disruption of the PD-(L)1 pathway helps induce immune tolerance and reinvigorates the innate antitumor capabilities of the immune system by upregulating T-cell activation and proliferation (68, 69). While the triggered auto-immunity exerts potent anti-tumor activity, it opens the door to a novel class of irAEs as well. IrAEs may manifest in any organ system but most frequently in the lung, skin, gut, endocrine glands, and liver (12). In the past few years, sarcoidosis and SLRs have been increasingly reported as a kind of rare irAE secondary to ICI treatment. Herein, we report the first case of hepatic SLR mimicking malignant metastasis in a NSCLC patient treated with the anti-PD-1 antibody toripalimab.

Our case adds to the growing bodies of literature of the world about ICIs-induced SLRs for malignancies. To the best of our knowledge, isolated hepatic granulomatous involvement has never been previously reported. It is noteworthy that liver injury has actually been recognized as a complication of ICI treatment (12), which manifests as hepatitis with liver enzymes or bilirubin elevation. But most of these patients were mild in severity without any radiologic findings or histological features except hepatic injury (70). However, in our case, the patient was asymptomatic and his laboratory tests were normal, the only clinical manifestation is the SLR found in liver radiologically presented as a single low-attenuation nodular lesion with dynamic contrast enhancement. He was firstly referred as a disease progression with liver metastasis, which was then correctly diagnosed as SLR depended on the histopathological findings. This is also the first reported case of granuloma formation induced by toripalimab, which showed efficiency and safety profiles comparable with other anti-PD-1 antibodies (11). The low incidence may probably be due to the limited use of this agent only for clinical trial. Based on the PC chemotherapy regimens along with toripalimab, our patient exhibited favorable therapeutic response at the presence of SLR, which also supports the positive relationship between SLRs and favorable therapeutic outcome. Similar with many previous cases reported, the SLR developed in this patient was gradually improved spontaneously. However, it is worth noting that although toripalimab, as the first domestic anti-PD-1 monoclonal antibody developed in China, has received conditional approvals for the treatments of melanoma, nasopharyngeal carcinoma and urothelial carcinoma in China, it is still restrictedly applied for clinical trials only in NSCLC patients all over the world. Though most of these preclinical studies in NSCLC are ongoing in China, we anticipate that further more international multicentric studies of toripalimab may start to provide more solid evidences for supporting its clinical use in the future.

In summary, we report a first case displaying isolated hepatic SLR in an NSCLC patient treated with toripalimab, and then we perform a brief review about the clinical features of sarcoidosis and SLRs associated to ICI treatment. With the increasing use of ICIs, we will see more similar cases in the near future. Misinterpretation of sarcoidosis and SLRs as disease progression would cause unnecessary second-line cancer treatments. It deserves the attention of clinicians on this kind of rare irAE, and histopathological evaluation of suspicious lesions developing upon immunotherapy, particularly in the setting of mixed responses, where it is necessary and critical to make sure and correct clinical decisions.

## Data Availability Statement

The original contributions presented in the study are included in the article/supplementary material. Further inquiries can be directed to the corresponding authors.

## Ethics Statement

The studies involving human participants were reviewed and approved by the Ethics Committee of Zhejiang Cancer Hospital. The patients/participants provided their written informed consent to participate in this study. Written informed consent was obtained from the individual(s) for the publication of any potentially identifiable images or data included in this article.

## Author Contributions

HYL and BCW had the idea for the article and provided the final approval of the version to be published. YXL performed the literature search, data analysis, and drafted the manuscript. WZ were involved in revising the manuscript critically for important scientific content. All authors listed have made a substantial, direct, and intellectual contribution to the work and approved it for publication.

## Funding

This work was supported by the National Natural Science Foundation of China (82102947 to HL), and the Zhejiang Provincial Natural Science Foundation of China (LQ22H160046 to HL).

## Conflict of Interest

The authors declare that the research was conducted in the absence of any commercial or financial relationships that could be construed as a potential conflict of interest.

## Publisher’s Note

All claims expressed in this article are solely those of the authors and do not necessarily represent those of their affiliated organizations, or those of the publisher, the editors and the reviewers. Any product that may be evaluated in this article, or claim that may be made by its manufacturer, is not guaranteed or endorsed by the publisher.
